# Chronic Coronary Artery Disease: Wall Disease vs. Lumenopathy

**DOI:** 10.3390/biom15020201

**Published:** 2025-01-31

**Authors:** Ioannis Paraskevaidis, Christos Kourek, Elias Tsougos

**Affiliations:** 1 Medical School of Athens, National and Kapodistrian University of Athens, 15772 Athens, Greece; chkourek@med.uoa.gr; 2 Department of Cardiology, Hygeia Hospital, 15123 Athens, Greece; cardio@tsougos.gr

**Keywords:** chronic coronary artery disease, myocardial ischemia, wall disease, lumenopathy

## Abstract

Acute and chronic coronary artery disease (CAD) are interconnected, representing two facets of the same condition. Chronic CAD exhibits a dynamic nature, manifesting as stable or acute ischemia, or both. Myocardial ischemia can be transient and reversible. The genesis of CAD involves diverse anatomical and functional mechanisms, including endothelial dysfunction, arteriolar remodeling, capillary rarefaction, and perivascular fibrosis, though no single factor explains its heterogeneity. Chronic CAD is often stable but may present as symptomatic or asymptomatic (e.g., in diabetes) and affect various coronary compartments (epicardial or microcirculation). This complexity necessitates a reappraisal of our approach, as pathophysiological mechanisms vary and often overlap. A comprehensive exploration of these mechanisms using advanced diagnostic techniques can aid in identifying the dynamic processes underlying CAD. The disease may present as obstructive or non-obstructive, stable or unstable, underscoring its diversity. The primary source of CAD lies in the arterial wall, emphasizing the need for research on its components, such as the endothelium and vascular smooth muscle cells, and factors disrupting arterial homeostasis. Shifting focus from arterial luminal status to the arterial wall can provide insights into the genesis of atheromatous plaques, enabling earlier interventions to prevent their development and progression.

## 1. Introduction

Recent guidelines define chronic coronary artery syndrome (CAS) as a condition arising from structural and/or functional alterations associated with chronic diseases of the epicardial coronary arteries and/or microcirculation, resulting in transient and reversible myocardial ischemia (MI) [1]. This definition emphasizes two critical aspects: chronicity and the reversibility of MI, both of which are influenced by the initiation and progression of atherosclerosis. Atherosclerosis is driven by multiple factors, including endothelial dysfunction, lipid accumulation, fibrosis, calcium deposition, and inflammatory activation [2].

**Figure 1:**
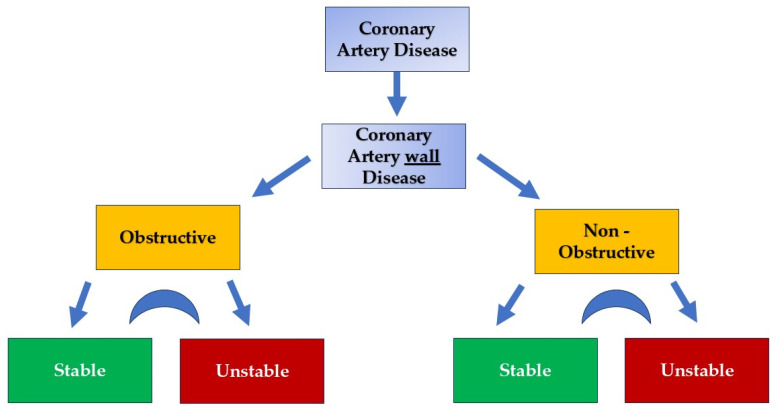
Coronary artery wall disease categorization. Stable and unstable stages can be presented in an interplay fashion.

Atherosclerosis is a slow and progressive process requiring several years for the development and maturation of atheromatous plaques. It can affect both large and medium-sized coronary arteries as well as the microvasculature. The disease may present as diffuse involvement without luminal stenosis or as localized focal lesions with significant intraluminal stenosis. Chronic coronary artery disease (CAD) differs fundamentally from acute coronary syndromes, necessitating a distinct diagnostic and therapeutic approach. This condition exhibits a dynamic profile, capable of manifesting in stable or acute forms, or transitioning between these states (Figure 1). Correspondingly, MI in chronic CAD is often transient and reversible. However, the precise mechanisms triggering this interplay remain unclear, warranting further investigation.

Multiple mechanisms contribute to the development of CAD, including endothelial dysfunction, arteriolar remodeling, capillary rarefaction, and perivascular fibrosis. Nevertheless, no single mechanism adequately explains the heterogeneity of the syndrome. Chronic CAD predominantly presents as a stable condition, which may be symptomatic or asymptomatic, with asymptomatic cases often observed in patients with diabetes mellitus. This disease can involve various coronary compartments, including the epicardial arteries and the microcirculation. Given the heterogeneity in pathophysiological mechanisms, it is imperative to adopt a nuanced approach, exploring the underlying pathophysiology and employing appropriate diagnostic techniques to identify the dynamic processes involved. This understanding will guide the development of more effective interventions.

It is important to note that MI can arise not only from luminal obstruction but also from diffuse arterial wall lesions that do not cause significant luminal narrowing [3]. Consequently, therapeutic strategies are often complex and challenging to determine. Decisions between optimal medical therapy and revascularization [4,5,6], as well as between percutaneous coronary intervention and coronary artery bypass grafting [7,8], require careful consideration. Furthermore, the choice of intervention based on the severity of MI remains a topic of ongoing debate [9,10]. These uncertainties highlight the need for continued discussion and research, as no single approach can be universally recommended.

It is also critical to recognize that CAD is fundamentally a disease of the arterial wall rather than the arterial lumen. This perspective underscores the importance of medical therapy and stringent risk factor management to mitigate the pathological effects on the coronary artery wall. These insights necessitate a reevaluation of the current understanding and therapeutic strategies [11,12,13].

## 2. Role of Coronary Microcirculation in CAD Development and Progression

Coronary microcirculation plays a pivotal role in maintaining myocardial perfusion and is a critical determinant of cardiac health. It consists of arterioles, capillaries, and venules that ensure adequate oxygen and nutrient delivery to cardiac tissues. The dysfunction of this compartment significantly contributes to the development and progression of CAD. Unlike the epicardial arteries, which serve as conduits, microcirculation is the principal site of vascular resistance and regulates coronary blood flow under varying physiological and pathological conditions [14,15].

In CAD, the microcirculation is affected by structural and functional changes that impair its ability to adapt to increased myocardial demand. Structural alterations, such as capillary rarefaction, arteriolar remodeling, and perivascular fibrosis, reduce the density and capacity of the vascular network. These changes are often exacerbated by chronic inflammation, endothelial dysfunction, and oxidative stress, which are hallmarks of CAD. Functional impairments, including reduced endothelium-dependent vasodilation, increased vascular stiffness, and enhanced microvascular resistance, further compromise myocardial perfusion [14,15,16].

Moreover, microvascular dysfunction can amplify the progression of CAD by creating a vicious cycle of ischemia, inflammation, and oxidative damage [16,17]. Impaired microcirculation limits the delivery of therapeutic agents to ischemic regions and hampers myocardial repair processes, thereby exacerbating disease severity. This dysfunction is particularly relevant in patients with diabetes mellitus, where microvascular complications are prevalent and contribute to atypical presentations of CAD, such as silent ischemia [18]. Additionally, microcirculatory abnormalities can persist even after the successful revascularization of epicardial arteries, leading to residual angina and poorer clinical outcomes [16].

Recent advances in diagnostic techniques, such as coronary flow reserve (CFR) measurement and index of microcirculatory resistance (IMR), have improved our understanding of microvascular function and its role in CAD [19,20]. These tools enable clinicians to identify microvascular dysfunction and tailor treatment strategies accordingly. Therapeutic approaches targeting microcirculation, including endothelial-protective drugs, anti-inflammatory agents, and lifestyle modifications, hold promise for mitigating the progression of CAD and improving patient outcomes. As such, further research into the mechanisms and therapeutic potential of coronary microcirculation is essential for advancing the management of CAD.

## 3. Myocardial Vasculature: Why and What to Open

The cardiac arterial vasculature is organized into three distinct compartments: the epicardial, the intermediate, and the capillary compartments. The epicardial arteries, with diameters ranging from 5 mm to 0.5 mm, serve as conduit arteries with minimal flow resistance [21]. Positioned superficially on the epicardium, their primary function is to facilitate blood flow to the deeper myocardial layers, ultimately reaching the microvascular compartment [21].

The intermediate compartment, consisting of vessels ranging from 0.5 mm to 0.1 mm in diameter, is further subdivided into a proximal part (0.5–0.15 mm) responsive to flow changes and a distal part (0.15–0.1 mm) sensitive to pressure variations. This compartment, comprising pre-arterioles and arterioles, plays a central role in regulating coronary blood flow [21,22]. The third compartment, the capillaries (less than 0.1 mm in diameter), is crucial for the physiological function of myocardial cells by performing three key functions: (1) supplying oxygen and nutrients, (2) removing metabolic waste products, and (3) facilitating repair and inflammatory processes [21,23].

The intramyocardial coronary circulation includes pre-arterioles, arterioles, and capillaries, collectively forming the coronary microcirculation. All components, except capillaries, consist of three layers: (a) the luminal layer, primarily comprising a single endothelial cell layer; (b) the media, composed of smooth muscle cells, elastic fibers, and collagenous tissue; and (c) the adventitia, consisting of connective tissue [21,22]. As vessels transition from the epicardial compartment to deeper myocardial regions, the thickness of the media and adventitia progressively decreases. Capillaries, which connect arteries to veins, consist of a single ultra-thin endothelial layer and serve as the primary delivery and exchange system for cardiac tissue. The capillaries transition into coronary veins (both greater and smaller venous systems), which collect metabolic byproducts and deoxygenated blood, returning them to the right atrium [21,22].

Given the above, the intramyocardial vasculature, particularly the arterioles and capillaries, represents the principal regulatory compartment of coronary circulation. This compartment is both a driver of blood flow and a pivotal functional entity in the circulatory system. In the context of acute CAS, it is well established that reopening the occluded epicardial artery is necessary to salvage myocardial tissue. However, this approach is less clear in the stable phase of chronic CAD [4,24].

In chronic CAS, the epicardial arteries, despite their primarily passive role, have been the focus of extensive research and industrial efforts aimed at determining the optimal method to reopen occluded arteries. Nevertheless, questions regarding why, when, and what to open remain unresolved. Reports suggest that even after percutaneous coronary intervention (PCI), residual angina and long-term stent-related events persist, leaving approximately 76% of the patients with what is described as an “illusion of revascularization” [25]. Furthermore, the debate continues on whether complete versus incomplete coronary revascularization is the superior approach, as complications and comorbidities associated with complete revascularization are difficult to predict.

In daily clinical practice, achieving optimal revascularization remains uncertain. In many cases, despite successful complete revascularization, elevated coronary microvascular resistance results in persistent symptoms. Notably, in stable patients with obstructive CAD, even after successful coronary angioplasty, a significant proportion exhibit low fractional flow reserve (FFR). This condition is associated with persistent angina symptoms and a poorer prognosis [26,27,28]. The elevated arterial resistance in the microcirculation leads to an increased FFR numerator (mean distal coronary pressure during maximal hyperemia), causing a false normalization of FFR values and obscuring the true physiological state.

## 4. Pathophysiologic Concerns and Questions

A significant number of patients with angiographically normal epicardial coronary arteries experience symptoms of MI and angina. This observation suggests that macrovascular and/or microvascular dysfunction, either independently or in combination, may underlie these conditions. Notably, MI can arise not only from fixed stenosis but also from diffuse epicardial arterial lesions, congenital anomalies, coronary epicardial vasospasm, and other factors. This highlights the need for a comprehensive diagnostic and therapeutic approach grounded in a deeper understanding of myocardial circulatory pathophysiology [1,3,29].

It is essential to focus on the various causes of MI and their clinical presentations. For instance, epicardial artery obstruction causes localized or global ischemia in large myocardial regions, whereas the microcirculation, through its vascular network, regulates adequate blood supply distribution. However, when microcirculatory dysfunction occurs, capillary transit time becomes unevenly distributed, leading to perfusion heterogeneity [15]. This disruption contributes to the development of the well-recognized primary or secondary coronary slow flow phenomenon, which in turn results in MI [30].

Patients with primary coronary slow flow frequently report chest pain (in 80–90% of the cases), experience hospital readmission in 33%, and have a 2.5% likelihood of adverse outcomes [30,31]. Primary coronary slow flow is an independent entity characterized by angiographically documented slow flow with less than 40% epicardial artery stenosis. In contrast, secondary coronary slow flow may result from coronary ectasia, stenosis, spasm, structural abnormalities, or other conditions that impair rheology or hemodynamic stability. Regardless of the etiology, affected patients commonly report angina without an apparent cause.

The relationship between angina symptoms and MI remains complex and controversial. Evidence suggests no direct correlation between the two, as demonstrated by studies involving patients with and without obstructive epicardial disease [32,33]. Nonetheless, a substantial number of cardiologists persist in equating angina with MI, advocating for revascularization as a means of improving symptoms by enhancing myocardial perfusion. While this approach assumes that addressing ischemia extent is critical, research indicates that ischemia severity itself does not predict outcomes. The COURAGE (Clinical Outcomes Utilizing Revascularization and Aggressive Drug Evaluation) trial demonstrated that while the severity of CAD correlates with adverse outcomes, ischemia severity alone, irrespective of its extent, does not [34]. Similarly, findings from the ISCHEMIA trial showed no association between ischemia severity and increased major cardiovascular events or all-cause mortality at a 4-year follow-up [10].

These findings emphasize the importance of addressing the root cause of chronic CAD rather than its secondary manifestations. Central to all forms of CAD is endothelial dysfunction. When the endothelium is exposed to biochemical, hormonal, or mechanical stressors, its homeostatic functions are disrupted, triggering processes that lead to atheromatous plaque formation [35,36]. Over time, the narrowing of the epicardial vessels occurs, accompanied by intramyocardial vasculature dysregulation, marking the onset of CAD [37].

The primary anatomical feature of epicardial CAD is the development of atheromatous plaques. These plaques begin forming in childhood and progressively worsen over time [38]. Initially confined to the coronary artery wall, they eventually protrude into the arterial lumen, restricting blood flow and causing oxygen supply–demand imbalance, which leads to ischemia [39]. This pathophysiological process, regardless of the underlying mechanism (e.g., stabilized intramural hematoma, coronary aneurysm), results from the toxic effects of various risk factors (e.g., smoking, dyslipidemia, hypertension, and diabetes) on the endothelium [2,40].

Arterial atherosclerosis is a multifactorial disease that can manifest as i) accelerated atherosclerosis, commonly seen in transplanted hearts, primarily due to immune mechanisms, and/or ii) endothelial and medial dysfunction [41].

Additionally, with aging, there is an increase in smooth muscle cells and interstitial collagen deposition in the intima, leading to the thickening of the vessel wall. Although this thickening may occur without the presence of atheroma, it can still impair blood flow and disrupt the exchange of substances [42].

### 4.1. Endothelium and Coronary Artery Atherosclerosis

**Figure 2:**
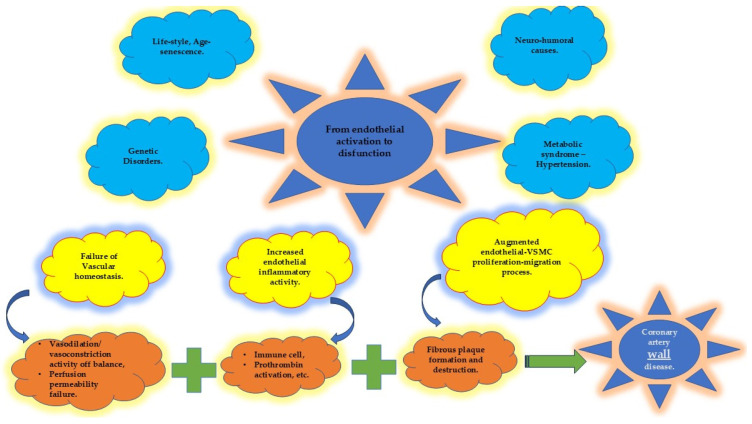
Endothelial homeostatic dysfunction (in yellow) due to multiple risk factors (in blue) leads to the disequilibrium of several endothelial properties (in orange) that, in turn, promote the beginning and progression of coronary artery wall disease. VSMC: vascular smooth muscle cell.

The endothelium, the innermost layer of blood vessels in the coronary circulatory system, is a monolayer that, along with collagen and elastic fibers, forms the intima. This luminal layer is in direct contact with blood and is contiguous with the medial layer of the vessel. Notably, the endothelium is a common feature of all the arteries and veins, regardless of their size. Serving as a barrier between blood and cardiac myocardial cells, the endothelium performs critical functions, acting as a sensor and transducer of signals to the other vascular wall layers. It regulates vascular homeostasis by responding to mechanical and metabolic stimuli, making it a pivotal factor in the genesis and progression of arterial atherosclerosis (Figure 2).

Hemodynamic forces, such as blood pressure and wall shear stress, may induce unstable tangential forces on endothelial cells. This results in morphological alterations (e.g., flattening, elongation, and volume changes) and functional disruptions (e.g., adhesion, proliferation, and loss of non-adhesive properties), creating a pro-atherosclerotic environment by promoting the migration of vascular smooth muscle and mononuclear cells [2,43,44].

Atherosclerosis is a multifaceted process initiated by the failure of endothelial homeostatic mechanisms. It is characterized by the formation of foam cells, lipid accumulation within macrophages and vascular smooth muscle cells, and the activation of inflammatory pathways. Fatty streak formation marks the early stage of this process, driven by hemodynamic factors that create turbulent flows. These flows compromise endothelial integrity and facilitate lipoprotein infiltration into the intima, thereby affecting the entire arterial wall [2,45,46].

Several genes, activated by mechanical forces exerted by blood flow, contribute to the progression of atherosclerosis. These include monocyte chemoattractant protein-1 [47], platelet-derived growth factors [48], eNOS, and platelet adhesion molecule-1 [49]. Additionally, pro-atherogenic genes, which may be either upregulated or downregulated, play a role in promoting endothelial dysfunction and atherosclerosis [2,50,51].

Risk factors such as aging, metabolic syndrome, hypertension, and oxidative stress further exacerbate endothelial dysfunction by disrupting homeostasis [51,52,53,54,55]. Consequently, the endothelium loses its barrier integrity, allowing the translocation of lipids, cell debris, and immune cells into the tunica intima. This leads to interactions between modified low-density lipoprotein (LDL), immune cells, and vascular smooth muscle cells, resulting in lipid accumulation within cells and the extracellular matrix [2]. This cascade of events activates inflammatory processes that perpetuate atherogenesis.

The earliest mechanical or chemical injury to the endothelium triggers the activation of vascular cell adhesion molecules (e.g., intercellular adhesion molecule-1 and E-selectin) on the endothelial surface [56]. This activation stimulates the production of chemokines and recruits myeloid cells from the peripheral circulation to the arterial intima. Monocytes transform into macrophages, while neutrophils release granule proteins that further promote cellular accumulation [57]. Macrophages engulf oxidized LDL, forming foam cells and fatty streaks. Oxidized LDL, reactive oxygen species, and the inflammatory response collectively lead to the deterioration and instability of atherosclerotic plaques [56].

**Figure 3:**
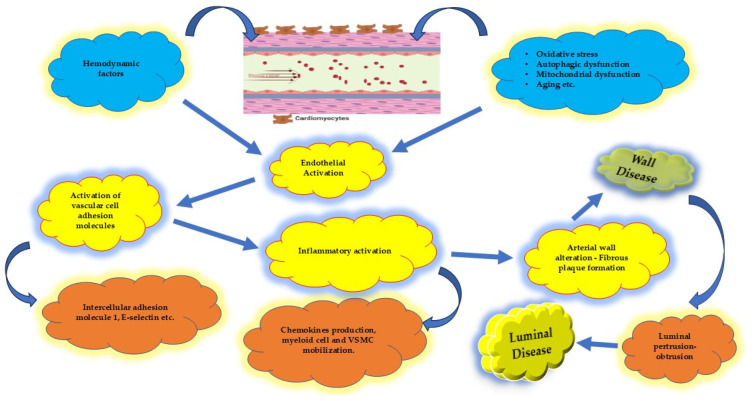
Sequence of progression when endothelial affection occurs. It begins as a wall disease whereas luminal disease represents the epiphenomenon of the wall disease. VSMC: vascular smooth muscle cell.

Given these insights, it may be more accurate to redefine the broad term “ CAD “ as “coronary artery wall disease” (Figure 2 and Figure 3). This concept encompasses both obstructive and non-obstructive disease, which can further be classified into stable and unstable wall disease categories [58] (Figure 1).

To mitigate coronary artery wall atherosclerosis, efforts should focus on modifying the influence of inherent and toxic-risk factors that contribute to endothelial dysfunction and arterial wall pathology [57,59,60] (Figure 2 and Figure 3). By addressing these upstream mechanisms, it may be possible to slow or reverse the progression of this complex disease process.

### 4.2. Systemic Inflammatory Disorders in CAD

Systemic inflammatory disorders play a crucial role in the development and progression of CAD. Chronic inflammation, driven by autoimmune diseases such as rheumatoid arthritis, systemic lupus erythematosus, and psoriasis, significantly increases the risk of CAD [61,62,63]. These conditions are associated with persistent immune activation and the production of pro-inflammatory cytokines, such as interleukin-6 (IL-6), tumor necrosis factor-alpha (TNF-α), and C-reactive protein (CRP), which exacerbate endothelial dysfunction and promote atherosclerotic plaque formation. Additionally, chronic inflammation contributes to plaque instability by enhancing matrix metalloproteinase activity, leading to the thinning of fibrous caps and an increased likelihood of rupture [62].

Beyond autoimmune disorders, low-grade systemic inflammation linked to obesity, metabolic syndrome, and diabetes also accelerates CAD progression [62,64]. Adipose tissue functions as an active endocrine organ, secreting adipokines that influence systemic inflammation and vascular health. For instance, elevated levels of leptin and resistin, combined with reduced adiponectin, promote atherogenesis by increasing oxidative stress and endothelial injury [65]. Understanding the interplay between systemic inflammatory disorders and CAD highlights the need for anti-inflammatory therapies.

### 4.3. Environmental Factors and Genetics in CAD

Environmental factors and genetic predisposition significantly influence the onset and progression of CAD. Lifestyle factors, including smoking, air pollution, and dietary habits, interact with genetic susceptibility to modulate cardiovascular risk [66]. Air pollution, particularly fine particulate matter (PM2.5), induces oxidative stress and systemic inflammation, which can impair vascular function and accelerate atherosclerosis [67]. Similarly, exposure to environmental toxins, such as heavy metals and polycyclic aromatic hydrocarbons, exacerbates oxidative damage and promotes endothelial dysfunction [68]. Addressing these environmental risk factors through public health initiatives can reduce CAD prevalence in vulnerable populations.

Genetic factors also play a fundamental role in CAD pathogenesis. Genome-wide association studies (GWASs) have identified numerous loci associated with CAD risk, including variants in genes involved in lipid metabolism (e.g., APOE and LDLR), inflammation (e.g., IL6R), and vascular homeostasis (e.g., NOS3) [69,70,71]. Epigenetic modifications, such as DNA methylation and histone acetylation, further modulate gene expression in response to environmental stimuli, creating a dynamic interplay between genetics and external factors [72]. The identification of polygenic risk scores (PRSs) has enabled clinicians to stratify patients based on genetic risk, paving the way for personalized preventive strategies [73]. Advancing our understanding of these genetic and environmental interactions is essential for developing targeted interventions that address the multifaceted nature of CAD.

### 4.4. Protecting the “Housekeeper”

The functional and structural integrity of the coronary artery wall relies predominantly on the health of the endothelium, often referred to as the “housekeeper” of arterial wall normality [50]. The endothelium serves as a key regulator of vascular homeostasis, controlling vascular tone, smooth muscle cell behavior, immune cell adhesion, and vascular inflammation [60]. The disruption of endothelial homeostasis represents the initial imbalance in the progression of coronary artery disease. This early dysfunction precipitates downstream events such as prothrombotic states, oxidative stress, endothelial cell transformation, apoptosis, and microvascular rarefaction. These processes collectively compromise the endothelial barrier, eventually contributing to organ dysfunction [51] (Figure 2 and Figure 3).

Is this sequence of events stable over time? Evidence suggests it is not. The endothelial response to harmful stimuli is dynamic, multifactorial, and varies in its progression timeline [74,75]. Therefore, efforts should be directed toward maintaining or restoring the functional and anatomical integrity of the arterial wall, with an emphasis on preventing or delaying the onset and progression of coronary artery wall disease.

Although the current medical treatments aim to improve endothelial function, they appear insufficient in fully halting the progression of arterial atherosclerosis. Lifestyle modifications and exercise training [76], alongside pharmacological interventions such as angiotensin-converting enzyme inhibitors (ACEis) [77], angiotensin receptor blockers (ARBs) [78], newer-generation β-blockers with vasodilatory properties [79], statins [80], and sodium–glucose cotransporter 2 inhibitors (SGLT2s) [81], have demonstrated encouraging and impactful results. However, these measures have not been successful in fully inhibiting the dynamic nature of the atherosclerotic process.

The complexity of endothelial dysfunction and its pivotal role in the pathogenesis of CAD underscore the need for continued innovation in therapeutic strategies. Future research should aim to identify approaches that not only address the manifestations of endothelial dysfunction but also target its root causes to slow or reverse the progression of coronary artery wall disease effectively.

#### 4.4.1. Vascular Homeostasis

Vascular homeostasis is a highly complex phenomenon, governed by the delicate balance between endothelium-derived relaxing factors (EDRFs) and endothelium-derived contracting factors (EDCFs) [82,83]. Key relaxing factors include prostaglandins, nitric oxide (NO), and endothelium-dependent hyperpolarization factors, while contracting factors include endothelin and angiotensin [84]. Disruptions to this equilibrium can arise from various cardiovascular risk factors, such as hypertension, diabetes, elevated low-density lipoprotein (LDL) cholesterol, and smoking, as well as other harmful stimuli like reactive oxygen species (ROS) and shear stress [85,86].

Nitric oxide, a potent vasodilator synthesized primarily by endothelial cells, plays a central role in maintaining vascular tone and homeostasis. However, in cases of endothelial dysfunction, NO bioavailability is significantly impaired, leading to the activation of pro-inflammatory and prothrombotic pathways. These alterations are critical contributors to the pathogenesis of atherosclerosis [50].

Although endothelial nitric oxide (eNOS) is a vital mediator of vascular health, efforts to enhance its production and activity have yielded mixed results. Despite the established importance of NO in vascular homeostasis, studies aimed at augmenting its levels or improving its bioavailability have been inconclusive and, in some instances, controversial. These findings underscore the complexity of NO’s role in the vascular system and highlight the need for further research to develop effective strategies for modulating NO activity and addressing endothelial dysfunction [87].

#### 4.4.2. Inflammatory Activity

Inflammatory activity in the arterial wall begins when endothelial protective mechanisms fail to counteract the adverse effects of hemodynamic, immune, and oxidative stress factors. These mechanisms are unable to prevent the oxidation of low-density lipoprotein (LDL), which enters the arterial wall through transcytosis from the plasma [2]. This triggers the release of inflammatory mediators by the endothelium, including chemokines, colony-stimulating factors, interleukins (e.g., IL-6, IL-8, and IL-1β), interferons, and monocyte chemoattractant protein-1. These mediators promote the adhesion and activation of pro-inflammatory monocytes (M1 phenotype) and neutrophils to the arterial walls. Concurrently, anti-inflammatory agents such as IL-35, IL-10, and M2 phenotype monocytes attempt to maintain homeostasis [88].

When this delicate balance is disrupted, endothelial dysfunction ensues, further impairing endothelial functional properties. This includes a reduction in antithrombotic function, increased expression of C-C motif chemokine ligand 2 and von Willebrand factor, degradation of the glycocalyx, diminished activation of protein C, and upregulation of tissue factor [50,88]. These changes exacerbate the inflammatory cascade and contribute to atherogenesis.

Additionally, the excessive production of free radicals, often linked to mitochondrial dysfunction, further compromises endothelial homeostasis, amplifying the processes driving arterial atherosclerosis [89].

Numerous attempts to modulate this inflammatory response have been made, primarily in experimental models, with promising but not yet definitive results [57,89]. Proposed strategies include targeting the chemokine–receptor network, ligand–receptor heteromer formation, and pathways that promote the resolution of inflammation. However, these approaches require further validation [57].

Experimental studies using endothelial-specific transgenic mice suggest that Nur77, a member of the orphan nuclear receptor 4A family, may represent a novel therapeutic target. Nur77 has been reported to attenuate endothelial dysfunction by enhancing nitric oxide production and activating antioxidant pathways in the vascular endothelium [90]. Despite these promising findings, further research is essential to understand and control the homeostatic status of the endothelium. Early intervention targeting the inflammatory processes may prevent the progression of arterial wall disease to its advanced stages, characterized by luminal pathology.

Addressing the resolution of inflammation is critical but remains highly complex. This calls for a multidisciplinary research effort. The current knowledge about anti-inflammatory drugs, genetic predispositions, circadian rhythms of immune responses, and specialized pro-resolving mediators should be incorporated into future investigations [57]. However, caution is needed to avoid the excessive suppression of immune responses, which could lead to significant complications and increased susceptibility to infections. The focus should remain on achieving a balance that effectively resolves inflammation without compromising immune function.

#### 4.4.3. Vascular Smooth Muscle Cells (VSMCs)

Vascular smooth muscle cells (VSMCs) have traditionally been considered pivotal in stabilizing atheromatous plaques [91]. However, emerging evidence indicates that the progression of atherosclerotic plaques is closely linked to the phenotypic plasticity of VSMCs, a process termed “phenotype switching” [92]. This refers to significant changes in the morphology, marker expression, and functionality of VSMCs. Specifically, VSMCs can transition to a synthetic phenotype, characterized by altered myofilament density, the diminished expression of contractile proteins, and the increased production of extracellular matrix (ECM) components [93].

During the early stages of atherogenesis, diffuse intimal thickening occurs, marked by the presence of synthetic VSMCs [94]. These cells are believed to originate from local medial VSMCs, although they differ from their precursors and significantly contribute to foam cell populations within plaques [95]. Remarkably, most foam cells in human coronary arteries are derived from VSMCs rather than monocytes, underscoring their critical role in plaque formation. Compared to medial VSMCs, synthetic VSMCs exhibit a higher organelle content, reduced expression of contractile proteins, and an increased capacity to retain apolipoproteins [93].

As atherosclerosis progresses, synthetic VSMCs enhance intimal thickening through ECM production and the accumulation of extracellular lipids [91,94]. This transitional phase, where the intima shifts from normality to pathology, depends on factors such as VSMC proliferation, phenotypic switching, apoptosis, lipid oxidation, and inflammation [93]. The synthetic VSMC-derived ECM also captures plasma-derived lipoproteins, which undergo modification, such as LDL oxidation, triggering immune responses and amplifying inflammation [96].

The progression of atherosclerosis involves the formation of a fibrous cap and a necrotic core composed of extracellular lipids, macrophages, and dead cells. Impaired efferocytosis (the process of clearing apoptotic cells, including VSMCs) leads to the accumulation of necrotic material, which activates damage-associated molecular patterns (DAMPs) and further stimulates the inflammatory cascade. Advanced plaques exhibit microcalcifications and calcium nodules that protrude into the arterial lumen. While these changes manifest as luminal disease, the underlying pathology lies within the arterial wall itself.

The role of VSMCs in the genesis, progression, and stabilization of atherosclerotic plaques is evident. This has prompted numerous studies to explore whether VSMCs can serve as therapeutic targets. Research into regulating phenotype switching, microRNA transcription, and epigenetic modifications has yielded promising results, but many questions remain unanswered [92,97].

To advance treatment strategies, a deeper understanding of VSMC proliferation, migration, and phenotype switching is essential. Future efforts should aim to develop therapeutic interventions that convert VSMCs from atherogenic to atheroprotective roles, integrate human genetic insights with VSMC biology, and potentially reverse or reprogram pathological VSMC behavior. Such breakthroughs could revolutionize the management of atherosclerosis and its associated complications.

## 5. Advanced Imaging Techniques in Atherosclerotic Disease

Recent advancements in imaging technologies have significantly enhanced our ability to understand the complex nature of atherosclerosis. These state-of-the-art modalities allow for the detailed visualization of both the structural and functional components of atherosclerotic plaques, providing invaluable insights into disease progression and risk assessment. For instance, intravascular ultrasound (IVUS) and optical coherence tomography (OCT) offer high-resolution imaging of the arterial wall, enabling the precise identification of plaque morphology, such as fibrous cap thickness and lipid core size [98]. Furthermore, near-infrared spectroscopy (NIRS) facilitates the detection of lipid-rich plaques, which are prone to rupture [99].

Non-invasive imaging techniques, such as computed tomography angiography (CTA) and magnetic resonance imaging (MRI), are increasingly used for assessing plaque burden, calcification, and vascular remodeling [100,101]. CTA has the advantage of detecting coronary artery calcification, which serves as a surrogate marker for overall atherosclerotic plaque burden, while advanced MRI techniques, including black-blood imaging and contrast-enhanced sequences, provide superior soft tissue characterization and can delineate plaque composition, such as distinguishing between fibrous and lipid-rich plaques. Positron emission tomography (PET), particularly when combined with CT or MRI, provides metabolic and molecular insights, including the evaluation of inflammatory activity within plaques. Tracer technologies, such as 18F-fluorodeoxyglucose (FDG) and newer macrophage-specific tracers, have enhanced the ability to localize and quantify active inflammation within atherosclerotic lesions [100,102].

Emerging modalities such as photoacoustic imaging [103,104] and molecular imaging [105,106] are also being investigated to provide even deeper insights into the cellular and molecular mechanisms of atherosclerosis. These techniques hold the potential to visualize the early markers of disease, such as endothelial dysfunction and neovascularization, which precede structural plaque changes.

Collectively, these imaging approaches not only enhance diagnostic accuracy but also hold promise for personalized therapeutic strategies by identifying high-risk plaques and monitoring the efficacy of interventions. Incorporating these technologies into routine clinical practice could bridge the existing gaps in our understanding of atherosclerotic disease, facilitate early detection, and improve patient outcomes by enabling timely and targeted interventions.

## 6. Clinical Events

As atheroma develops within the arterial lumen, two distinct clinical scenarios can emerge: acute events or chronic conditions. The determining factor between these scenarios is plaque stability. Acute events are most commonly caused by coronary arterial thrombosis due to plaque rupture, but they can also result from plaque erosion or thrombosis on calcified nodules. However, silent thrombi frequently form and are often repaired or healed, thereby averting progression to acute events. This observation introduces a third scenario characterized by the alternation between stable and unstable states. Chronic coronary artery wall disease exhibits a dynamic profile, with periods of stability interrupted by episodes of instability, occurring in an interplay-like manner.

Clinical efforts should prioritize identifying vulnerable plaques, stabilizing them, and preventing their transition to an unstable state. Encouragingly, routine clinical practice has demonstrated a decline in all forms of acute coronary artery disease, attributed to effective risk factor management, including hypertension control, LDL-lowering therapies, and smoking cessation. Similarly, rehospitalization rates for chronic coronary disease have declined, indicating that drug-based medical therapy can mitigate the dynamic nature of the disease and reduce its interplay characteristics [93,107,108].

## 7. Identification of the Source and Therapeutic Actions

The cornerstone of identifying and treating arterial wall disease lies in recognizing endothelial dysfunction and the presence of unstable or vulnerable plaques. Both invasive techniques (e.g., intravascular ultrasound, optical coherence tomography, near-infrared spectroscopy, and ultrasound-based virtual histology) and non-invasive modalities (e.g., computed tomography coronary angiography, positron emission tomography, and magnetic resonance imaging) are utilized for this purpose. However, these methods have inherent limitations, often providing incomplete or conflicting information [107,108,109].

Advancements in imaging techniques are needed to improve spatial and temporal resolution and enhance tissue characterization capabilities. These improvements should aim to identify arterial wall disease by offering detailed insights into vascular homeostasis, endothelial inflammatory activity, and vascular smooth muscle cell (VSMC) function. Integrating imaging data with clinical assessments, novel biomarkers (e.g., C-reactive protein, insulin, osteopontin, osteoprotegerin, copeptin, TGF-β cytokines, homocysteine, myeloperoxidase, and soluble intercellular adhesion molecule-1), and personal genetic information is essential for more accurate diagnosis and management [110].

Once unstable or vulnerable plaques are identified, new therapeutic approaches should be considered. The current treatments, including angiotensin-converting enzyme inhibitors (a-MEA), β-blockers, antihypertensive drugs, and sodium–glucose cotransporter 2 inhibitors (SGLT2s), improve endothelial function. LDL-cholesterol control through statins, ezetimibe, and PCSK9 inhibitors has demonstrated substantial endothelial-protective effects. However, other approaches, such as peroxisome proliferator-activated receptor-α modulators (e.g., niacin) and cholesteryl ester transfer protein inhibitors, have not shown comparable benefits [111,112].

Anti-inflammatory therapies have yielded mixed results. Some studies report no change in the total atheroma volume [113], while others indicate significant improvements in plaque morphology [114]. The inhibition of IL-1β with canakinumab effectively reduces inflammation, but the residual inflammatory risk associated with IL-18 and IL-6 remains. Targeting chemokines, chemokine receptors, or heteromeric chemokine–receptor interactions is a promising but underexplored area [57]. The circadian rhythms of immune cell activation and the timing of interventions are emerging considerations but require validation in larger clinical trials [115,116,117].

Other innovative approaches, such as hyperbaric oxygen therapy (promoting immunomodulation, stem cell regeneration, and antioxidant effects) [118] and hemadsorption (removing inflammatory toxic products in critically ill patients) [119], have also been proposed. Table 1 demonstrates the underlying mechanisms in CAD and lists potential pharmacological approaches to overcome them.

### 7.1. Preventing Inflammation and Beyond

**Figure 4:**
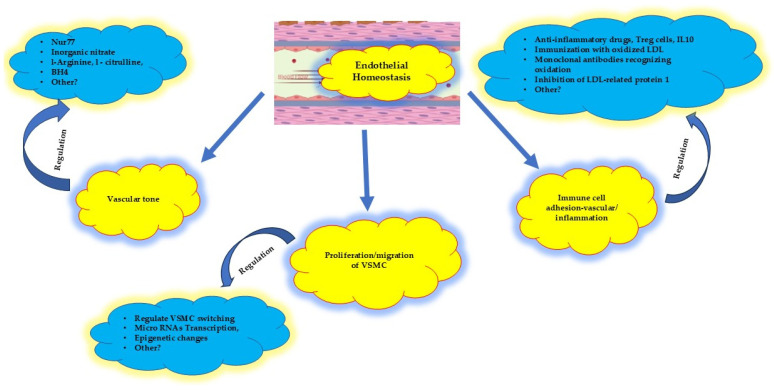
Proposed therapeutic targets at the early stage of atherosclerosis, at the very beginning of endothelial homeostatic dysfunction. The vascular tone, the proliferation/migration of VSMC cells, and the immune adhesion cell activation might be regulated. VSMC: vascular smooth muscle cells; Nur77: nuclear receptor subfamily 4 group A member 1; Tetrahydrobipterin (BH4): enzymatic cofactor; Treg: T immune regulator cells. See text for more details; IL10: interleukin 10; LDL: low-density lipoprotein.

The current anti-inflammatory strategies primarily focus on stabilizing the existing atherosclerotic plaques, which is critical but insufficient. Greater emphasis should be placed on mitigating pro-inflammatory signals early in the disease process. Polyunsaturated fatty acids, regulatory T cells, and IL-10 inhibit immune cell activation and suppress adhesion molecule expression, preserving endothelial integrity and halting inflammation before it fully manifests (Figure 4) [59,60,120,121]. Experimental models using immunization with oxidized LDL, monoclonal antibodies targeting oxidation, and inhibition of low-density lipoprotein receptor-related protein-1 have shown promise, but these approaches require further validation [122].

Another potential therapeutic avenue involves regulating transcription and microRNAs to protect endothelial cells from the proatherogenic effects of oxidized phospholipids, thereby addressing dysfunction at its earliest stages [123].

### 7.2. VSMC Modulation

Controlling VSMC phenotypic modulation is another critical area of research. Preventing the transition of VSMCs from a contractile to a proliferative phenotype could limit disease progression. Experimental studies utilizing basic fibroblast growth factor and heparin-binding epidermal growth factor-like growth factor have shown positive results, but more research is needed to confirm these findings and develop clinical applications [122].

Efforts to integrate these novel approaches into personalized treatment strategies could significantly advance the prevention and management of coronary artery wall disease and its complications.

## 8. Limitations and Future Directions in Genetic Research in CAD

While this study provides significant insights into chronic CAD and its underlying mechanisms, several limitations should be acknowledged. First, the ability to draw causal inferences regarding the progression of CAD and its relationship with various pathophysiological mechanisms is limited in the manuscript. Additionally, the reliance on current imaging modalities, though advanced, may not fully capture early subclinical changes in the coronary microcirculation or arterial wall. Furthermore, the study’s focus on specific diagnostic techniques and therapeutic strategies might limit its generalizability across diverse patient populations with varying disease presentations.

Future research into the genetic factors underlying CAD involves both prevention and management strategies. Large-scale studies, such as genome-wide association studies (GWASs) and whole-genome sequencing (WGS), are expected to identify novel risk alleles associated with CAD [124]. These efforts will deepen our understanding of the genetic architecture of the disease and uncover new biological pathways involved in its progression. The integration of polygenic risk scores (PRSs) into clinical practice could enable the identification of individuals at high genetic risk for CAD, even before the onset of symptoms [73]. Such individuals would benefit from targeted preventive measures, including lifestyle interventions and pharmacological therapies, tailored to their specific risk profiles.

In addition to genetic insights, the use of biochemical markers for early detection represents a promising avenue for research. Markers such as high-sensitivity C-reactive protein (hs-CRP), lipoprotein (a), and the emerging biomarkers of endothelial dysfunction and inflammation could complement genetic tools in identifying at-risk populations [125]. Combining genetic and biochemical data with advanced imaging techniques and risk prediction models would further enhance the precision of early detection strategies [126]. These approaches would enable healthcare providers to implement personalized preventive measures, reduce disease burden, and improve outcomes in populations susceptible to CAD.

## 9. Conclusions

Coronary artery lumen obstruction should be regarded as an epiphenomenon rather than the disease itself. The true source of the disease resides within the wall of the coronary arteries. Thus, our efforts and research must prioritize understanding and targeting the structural and functional components of the arterial wall, including the endothelium and vascular smooth muscle cells (VSMCs), as well as the factors that disrupt arterial homeostasis. Future strategies should explore novel approaches that complement the existing therapies by addressing the pro-inflammatory cascade in its early stages, aiming to control rather than abolish the inflammatory process. Additionally, integrating these approaches with insights from personal genetic backgrounds may provide more individualized and effective interventions. The focus should shift from merely treating advanced stages of atheromatous plaque to recognizing and intervening at the earliest stages of its genesis, or even preventing its formation altogether. Early intervention, rooted in understanding the underlying mechanisms of disease initiation, offers the most promising path toward reducing the burden of coronary artery disease. The earlier, the better.

## Figures and Tables

**Table 1:** Underlying mechanisms in CAD and pharmacological strategies.

Underlying Mechanism in CAD	Pharmacological Strategy
Endothelial dysfunction	ACE inhibitors, ARBs, statins, and NO donors
Inflammation	Anti-inflammatory therapies including IL-1β inhibitors, TNF-α blockers, etc.
Oxidative stress	Antioxidants including vitamin E, Nrf2 activators, etc.
Vascular smooth muscle cells dysfunction	Drugs targeting VSMC phenotype switching including TNF-β inhibitors, microRNA modulators, etc.
Plaque instability	MMP inhibitors and PCSK9 inhibitors
Microvascular dysfunction	Endothelial protective drugs, vasodilators, and Ca blockers
Lipid accumulation and foam cell formation	Statins, PCSK9 inhibitors, and therapies reducing LDL oxidation

CAD, coronary artery disease; ACE, angiotensin-converting enzyme; ARB, angiotensin receptor blockers; NO, nitric oxide; IL, interleukin; TNF, tumor necrosis factor; Nrf2, nuclear factor erythroid 2-related factor 2; VSMC, vascular smooth muscle cells; MMP, matrix metalloproteinases; PCSK9, proprotein convertase subtilisin/kexin type 9; Ca, calcium; LDL, low-density lipoprotein.
